# Frailty in cerebellar ischemic stroke—The significance of temporal muscle thickness

**DOI:** 10.3389/fneur.2023.1193685

**Published:** 2023-09-26

**Authors:** Daniel Dubinski, Sae-Yeon Won, Isabell Mattes, Svorad Trnovec, Bedjan Behmanesh, Daniel Cantré, Peter Baumgarten, Nazife Dinc, Juergen Konczalla, Matthias Wittstock, Thomas M. Freiman, Florian Gessler

**Affiliations:** ^1^Department of Neurosurgery, University Medicine Rostock, Rostock, Germany; ^2^Institute of Diagnostic and Interventional Radiology, Pediatric Radiology and Neuroradiology, University Medicine Rostock, Rostock, Germany; ^3^Department of Neurosurgery, University Hospital, Schiller University Jena, Jena, Germany; ^4^Department of Neurosurgery, Goethe-University Hospital, Frankfurt am Main, Germany; ^5^Department of Neurology, University Medicine Rostock, Rostock, Germany

**Keywords:** cerebellar stroke, ischemic stroke, posterior fossa, temporal muscle thickness, sarcopenia, out-come, risk factors

## Abstract

While comprising only 2% of all ischemic strokes, cerebellar strokes are responsible for substantial morbidity and mortality due to their subtle initial presentation and the morbidity of posterior fossa swelling. Furthermore, low temporal muscle thickness (TMT) has recently been identified as a prognostic imaging parameter to assess patient frailty and outcome. We analyzed radiological and clinical data sets of 282 patients with cerebellar ischemic stroke. Our analysis showed a significant association between low TMT, reduced NIHSS and mRS at discharge (*p* = 0.035, *p* = 0.004), and reduced mRS at 12 months (*p* = 0.001). TMT may be used as a prognostic imaging marker and objective tool to assess outcomes in patients with cerebellar ischemic stroke.

## Introduction

The cerebellum is a complex and multifunctional structure that is essential for a wide range of motor and cognitive functions. Moreover, the cerebellum is also engaged in a variety of processes, such as the processing of sensory data, the control of eye movements, the control of balance and posture, and the coordination of motions (1). In terms of memory formation, the cerebellum is involved in the acquisition and retention of motor skills through long-term changes in synaptic connections which result in the formation of motor memory (2, 3).

A cerebellar stroke is a type of cerebrovascular event involving the posterior cranial fossa. Arterial obstruction leads to impaired blood and oxygen delivery following impaired perfusion which leads to deficits in motor and balance control (4). However, presenting symptoms are often non-specific and overlap with other neurologic, cardiovascular, gastrointestinal, and systemic conditions which often makes rapid diagnosis difficult (5). Surgical decompressive suboccipital craniotomy/craniectomy with or without removal of the infarcted tissue is indicated in cases with a space-occupying cerebellar infarction to prevent coma and death. Well-established risk factors for the development of cerebellar stroke include vasculopathies such as atherosclerosis or arterial dissections, specifically of the vertebral arteries (6). Data on functional outcomes after cerebellar stroke are sparse, and the reasons for poor outcomes remain unclear (7–9). Among the discussed causes is the strength-sapping rehabilitation process that is dependent on a vigorous nutritional condition (10).

However, the current topic of great interest in the scientific community is frailty. Frailty defines a state of increased vulnerability, decreased physiological reserves, and reduced capacity to withstand stressors. It is often associated with aging and can result from a combination of factors such as chronic diseases, functional decline, cognitive impairment, and nutritional deficiencies. Frail individuals are at a higher risk of adverse health outcomes, including falls, hospitalization, disability, and mortality due to their decreased ability to recover from illness or other challenges.

On the other hand, the radiological measurement of skeletal muscle mass was recently introduced as an objective parameter for outcomes in brain tumor, subdural hematoma, and stroke patients (11–13). Cranial computed tomography (CT) scans and magnetic resonance imaging (MRI) are routinely performed on cerebellar stroke patients; therefore, temporal muscle thickness (TMT) measurement is easy to perform and quick to implement. We investigated TMT as a parameter to evaluate patient frailty and investigated its role as a novel prognostic marker for cerebellar stroke outcomes.

## Methods

### Study design

All patients admitted to the neurosurgical department of the authors' institutions between August 2016 and June 2021 with the diagnosis of a cerebellar stroke were included in the analysis. The inclusion criteria were available CT/MRI scans and the age of patients aged 18 years and above. The exclusion criteria were the presence of structural lesions, hemorrhagic transformation, or tumor, as well as the lack of radiological data or hospital discharge in <24 h after admission. CT and MRI scans are essential techniques for diagnosing and monitoring cerebellar infarction and the lack thereof was chosen as an exclusion criterion to ensure high quality and minimize bias. The exclusion criteria for discharge in <24 h after admission was chosen since longer hospital stays are necessary to provide thorough monitoring as well as to identify any potential issues or additional negative outcome parameters. Patient characteristics and medical data were collected using the institutional electronic database. For this retrospective analysis, ethical approval was obtained from the Ethics Committee of the University Medicine Rostock, Germany (Identification number: A 2021-0112). As a non-interventional monocentric study, patient consent was waived. Investigated medical record parameters included age at admission, sex, GCS, NIHSS (National Institutes of Health Stroke Scale) and mRS (Modified Rankin Scale) at admission, anticoagulation status, preexisting conditions, radiological parameters such as infarct and cerebellar volume, clinical course, and status at discharge and at 12 months.

### Image analysis

**Figure 1:**
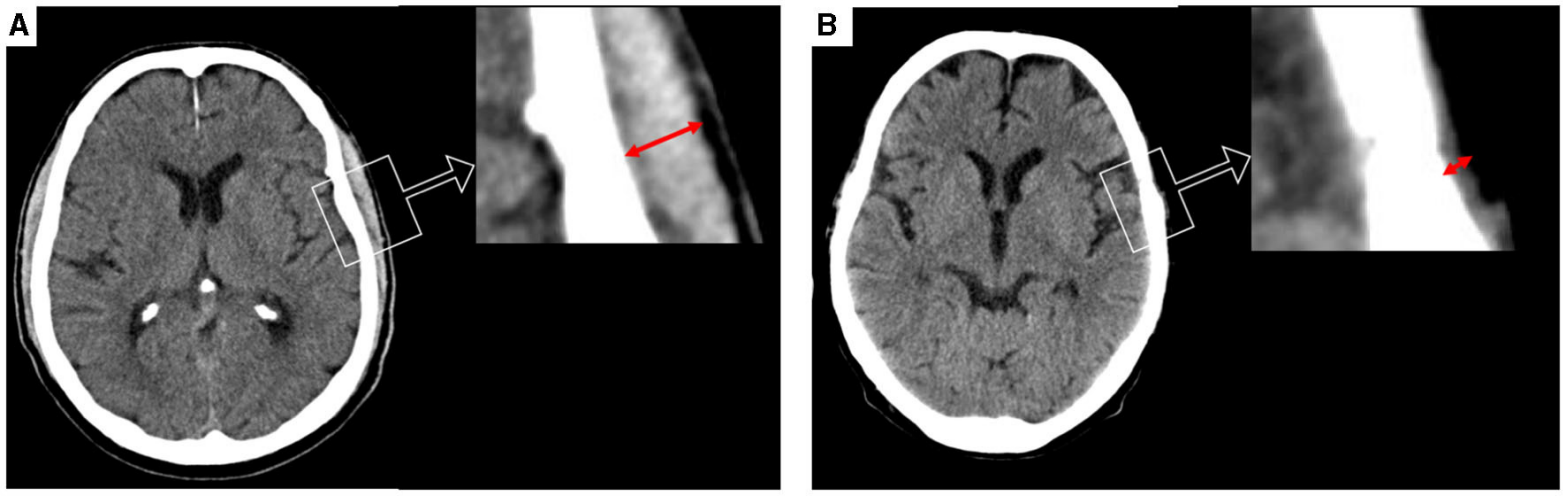
Representative cases for the assessment of temporalis muscle thickness (TMT) on cranial CT scan shown in red arrows. **(A)** TMT measurement on axial images of a patient with high TMT (bilateral median TMT = 10.5 mm), and **(B)** a patient with a low TMT on axial CT scan analysis (bilateral median TMT = 1.5).

TMT on T2-weighted MR images and CT scans were analyzed using the PACS software Jivex^®^ v5.2 (VISUS Technology Transfer GmbH, Germany). The volume of cerebellar stroke was calculated with the use of region-of-interest measurement via the Brainlab software (Brainlab^®^ AG, München, Germany). Image analysis was performed by two neurosurgeons (D.D. and S.Y.W.) who were blinded to the patient's medical data. A representative analysis is displayed in Figure 1. The plane with maximum TMT was measured on the left and the right side separately in each patient, and each side was summed up and divided by two, resulting in a median TMT per patient. The median TMT of overall patients was 5.5 mm (range 3.5–12.5). The median value was set as the critical point and divided the cohort into the “low TMT” and the “high TMT.”

### Statistics

Data analysis was performed using GraphPad Software 2023 (GraphPad Software, San Diego, California, USA). For patient characteristics, descriptive statistics was used. Fisher's exact test was used for the comparison of continuous parameters between the cohorts. For categorical parameters, the Wilcoxon/Mann–Whitney test was used. To assess the impact of the variables, odds ratios (ORs) with 95% confidence intervals (CIs) were calculated. Results with a p-value of ≤0.05 were considered to be statistically relevant.

## Results

### Study population and descriptive analysis

**Figure 2:**
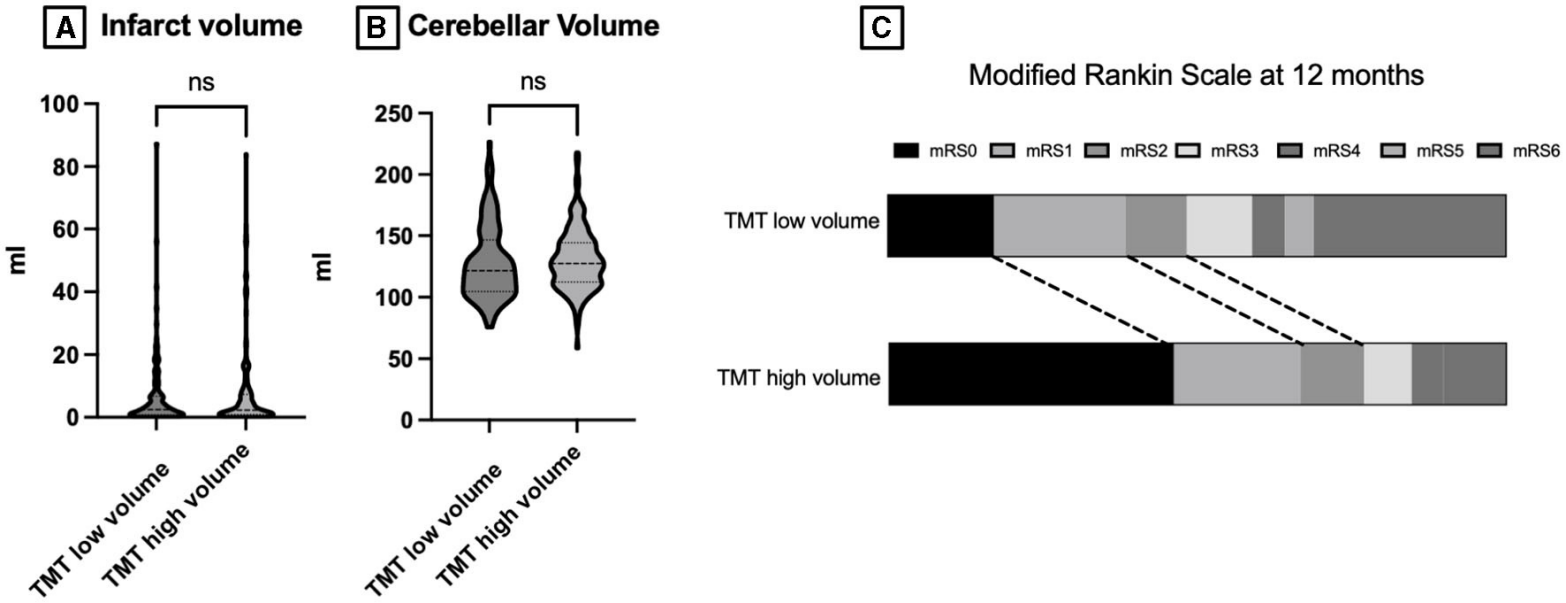
**(A)** Plotted infarct volume in ml stratified according to low vs. high temporal muscle thickness. **(B)** Plotted cerebellar volume in ml stratified into low vs. high temporal muscle thickness. **(C)** mRS at 12 months stratified into low vs. high temporal muscle thickness. TMT, temporal muscle thickness; mRS, modified Rankin scale; ns, not significant.

A total of 287 patients with cerebellar stroke were screened for inclusion. After five patients were excluded due to missing radiological data, a total of 282 patients were included in the final analysis. Of the 282 cerebellar stroke patients, 128 patients were men (45%) with a median age of 71 years (IQR: 64–81). The median NIHSS score at admission was 5 (IQR: 2–6), GCS 15 (IQR: 15), and 41 patients had a positive history of anticoagulation treatment including antiplatelet therapy (15%). Regarding preexisting comorbidities, 222 patients had a positive history of hypertension (79%), 67 patients had atrial fibrillation (24%), 76 patients had diabetes mellitus (27%), and 48 patients had coronary heart disease (17%) (Table 1). The median infarct volume was 7 ml (IQR 0–7), the cerebellar volume 129 ml (IQR: 109–144), and the temporal muscle thickness 5 mm (IQR: 0–7). The exclusive analysis of a single TMT site did not show any significant difference compared to the median used here. Surgical treatment was performed in 13 cases (4%), lysis in 44 cases (16%), thrombectomy in 18 cases (6%), and conservative management in 207 cases (73%). Median NIHSS at discharge was 6 (IQR: 0–7), and median mRS was 3 (IQR: 1–3). Of 229 patients at 12 months follow-up, the median mRS was 3 (Q1–Q3: 0–5) (see Figure 2).

**Table 1:** Demographics, management, and outcome data.

**Patient characteristics**	**(*n* = 282)**
**Sex**	
Male, *n* (%)	128 (45)
**Age**, median (IQR)	71 (64–81)
**Admission status**	
NIHSS at admission, median (IQR)	5 (2–6)
GCS at admission, median (IQR)	15 (15)
Anticoagulation, *n* (%)	41 (15)
**Preexisting comorbidities**	
Hypertension, *n* (%)	222 (79)
Atrial fibrillation, *n* (%)	67 (24)
Diabetes mellitus, *n* (%)	76 (27)
Coronary heart disease, *n* (%)	48 (17)
**Radiological parameters**	
Infarct volume median, ml (IQR)	7 (0–7)
Cerebellar volume, median, ml (IQR)	129 (109–144)
Temporal muscle thickness median (IQR)	5 (0–7)
**Treatment**	
Operative, *n* (%)	13 (4)
Thrombolytic therapy, *n* (%)	44 (16)
Thrombectomy, *n* (%)	18 (6)
Medical management, *n* (%)	207 (73)
**Outcome**	
NIHSS at discharge, median (IQR)	6 (0–7)
mRS at discharge, median (IQR)	3 (1–3)
mRS at 12 months (*n* = 229), median (IQR)	3 (1–3)

### Characteristics and admission status in cerebellar stroke patients according to temporal muscle thickness

In our univariate and multivariate analyses, increased patient age showed an association with low TMT (median of 85 vs. 69 years; p = 0.001 in univariate analysis and p = 0.043 in multivariate analysis). Furthermore, univariate and multivariate analyses showed a significant association between low TMT and increased NIHSS score at admission: 4 vs. 2; p = 0.001 in univariate analysis and p = 0.001 in multivariate analysis. Furthermore, anticoagulation therapy at admission was significantly associated with low TMT in the univariate analysis: 18% in the low TMT cohort vs. 7% in the high TMT cohort; p = 0.032. Patient sex and GCS at admission were not significantly associated with TMT (Tables 2, 3).

**Table 2:** Univariate analysis of juxtaposed characteristics according to TMT in cerebellar stroke.

**Patient characteristics (*n* = 282)**	**Median TMT**		**Univariate analysis**		
	**Low** ***n*** = **192**	**High** ***n*** = **90**	**OR**	**95% CI**	***p*** **-value**
**Sex**					
Male, *n* (%)	85 (44)	69 (76)	4.1	2.34–7.28	n.s.
**Age**, median (IQR)	78 (67–83)	68 (59–75)		12.81–19.12	0.001
**Admission status**					
NIHSS at admission, median (IQR)	4 (2–6)	2 (1–4)		1.07–2.93	0.001
GCS at admission, median (IQR)	15 (14,15)	15 (15)		0.21–0.21	n.s.
Anticoagulation, *n* (%)	35 (18)	7 (7)	2.6	1.12–6.20	0.032
**Preexisting comorbidities**					
Hypertension, *n* (%)	157 (82)	65 (72)	1.7	0.95–3.10	n.s.
Atrial fibrillation, *n* (%)	56 (29)	11 (12)	2.9	1.46–5.97	0.003
Diabetes mellitus, *n* (%)	52 (27)	24 (27)	1.0	0.58–1.79	n.s.
Coronary heart disease, *n* (%)	36 (19)	12 (13)	1.5	0.73–3.04	n.s.
**Radiological parameters**					
Infarct volume median, ml (IQR)	2 (0–7)	2 (0–8)		1.61–4.21	n.s.
Cerebellar volume, median, ml (IQR)	122 (107–145)	128 (112–143)		2.78–14.78	n.s.
**Treatment**					
Operative, *n* (%)	9 (5)	4 (4)	1.0	0.31–3.52	n.s.
Thrombolytic therapy, *n* (%)	27 (14)	17 (19)	0.7	0.36–1.36	n.s.
Thrombectomy, *n* (%)	12 (6)	6 (7)	0.9	0.33–2.57	n.s.
Medical management, *n* (%)	144 (75)	63 (70)	1.2	0.73–2.24	n.s.
**Outcome**					
NIHSS at discharge, median (IQR)	2 (0–5)	1 (0–3)		0.07–1.93	0.035
mRS at discharge, median (IQR)	3 (1–4)	2 (1–3)		0.32–1.68	0.004
mRS at 12 months, median (IQR)	3 (1–6)	1 (0–2)		0.92–3.08	0.001

OR, odds ratio, IQR; interquartile range; GCS, Glasgow Coma Scale; NIHSS, National Institutes of Health Stroke Scale; mRS, Modified Rankin Scale; ns, not significant.

**Table 3:** Multivariate analysis of juxtaposed characteristics according to TMT in cerebellar stroke.

**Patient characteristics (*n* = 282)**	**Median TMT**		**Multivariate**		
	**Low** ***n*** = **192**	**High** ***n*** = **90**	**OR**	**95% CI**	***p*** **-value**
**Age**, median (IQR)	78 (67–83)	68 (59–75)	1.2	1.01–1.04	0.043
**Admission status**					
NIHSS at admission, median (IQR)	4 (2–6)	2 (1–4)	2.5	0.87–4.01	0.001
Anticoagulation, *n* (%)	35 (18)	7 (7)			n.s.
**Preexisting conditions**					
Atrial fibrillation, *n* (%)	56 (29)	11 (12)			n.s.
**Outcome**					
NIHSS at discharge, median (IQR)	2 (0–5)	1 (0–3)	2.1	1.05–1.26	0.001
mRS at discharge, median (IQR)	3 (1–4)	2 (1–3)	2.2	0.86–1.71	0.032
mRS at 12 months, median (IQR)	3 (1–6)	1 (0–2)	3.4	1.21–1.67	0.001

OR, odds ratio; IQR, interquartile range; GCS, Glasgow Coma Scale; NIHSS, National Institutes of Health Stroke Scale; mRS, Modified Rankin Scale; ns, not significant.

### Preexisting comorbidities and radiological parameters stratified according to patients' TMT

In the univariate analysis, a history of atrial fibrillation was significantly associated with reduced TMT (29% vs. 12%; p = 0.003). Additionally analyzed factors including hypertension, diabetes mellitus, and coronary heart diseases showed no statistically significant association with patient TMT. In terms of infarct volume and cerebellar volume, no significant association with TMT was identified.

### Treatment and outcome stratified according to TMT

Neither surgical therapy, thrombolytic therapy, thrombectomy, or medical management demonstrated a significant association with TMT. However, univariate and multivariate analyses showed a significant association with increased NIHSS scores at discharge (2 in the low TMT cohort vs. 1 in high TMT; p = 0.032 and 0.001). Furthermore, the modified Rankin scale at discharge was significantly associated in univariate and multivariate analyses with patients' TMT (3 in low TMT vs. 2 in patients with high TMT; p = 0.004 and p = 0.003, respectively). In the 12-month follow-up, univariate and multivariate analysis confirmed the increased mRS score with (3 in the low TMT vs. 1 in the high TMT cohort: p = 0.001 and p = 0.001, respectively).

## Discussion

This study investigates the role of TMT on conventional cranial CT and MRI scans performed on patients with cerebellar ischemic stroke. The major finding is the significant association between low TMT and decreased admission, as well as discharge and 12-month follow-up status. The findings of this study suggest that TMT may represent an objective parameter with prognostic value, which is novel for cerebellar ischemic stroke patients.

Frailty and the often synonymously used term of sarcopenia are becoming increasingly important in the scientific community due to their reliable prognostic value in brain tumor patients and increasingly extended to other neurosurgical conditions such as neurotrauma (14, 15). For instance, Namgung et al. recently showed the association between temporal muscle mass and early cognitive function in patients with mostly supratentorial acute ischemic stroke. The analysis showed that TMT was an independent predictor of early post-stroke cognitive function, stratified by the MoCA score (16).

However, an objective measurement of sarcopenia in clinical practice is hampered as it is usually verified through muscle function tests such as the gait speed test and/or the grip strength test, which often cannot be performed in ischemic stroke patients due to frequently present disturbances of consciousness and/or neurologic deficits (13, 17, 18). Another fact is that clinical deficits after isolated cerebellar stroke are subtle and poorly represented by the NIHSS, and the demand for alternative predictive parameters in this cohort is therefore crucial (19).

In our cohort, patient age was significantly associated with reduced TMT both in the univariate and multivariate analyses. We considered the possibility of bias with respect to the fact that older patients are intrinsically at higher risk for poor clinical outcomes and that this fact could undermine the role of TMT. However, even if accounting for the fact that muscle mass could be affected by age, sarcopenia as such is affected by several additional age-unrelated factors such as hormonal changes or inflammation. Furthermore, in contrast to the classical predictors of outcome in supratentorial stroke, several studies reported no association between patient age and clinical outcome (4, 6). Furthermore, other studies could not show a significant association between patients' age and TMT (Katsuki et al.). In summary, we value the age-related correlation between TMTs but insist on the value of TMT as an independent risk factor for poor outcomes in ischemic stroke patients. However, for a detailed classification of the data presented here, TMT values should be measured for a healthy comparison group in future prospective studies.

In terms of the analyzed preexisting comorbidities, only atrial fibrillation showed an association with reduced TMT, a finding that was statistically non-significant in the multivariate analysis. The role of comorbidities in the outcome after cerebellar stroke remains ambiguous, and several studies failed to observe a significant role of comorbidity in the prediction of functional outcomes after acute cerebellar infarctions (20). In our opinion, this fact undermines the significance of the TMT by largely excluding the preexisting conditions as bias.

With regard to the correlation between TMT and outcome in stroke patients, a recent analysis by Sakai et al. reported the association between reduced TMT and ischemic cerebral stroke-related dysphagia in 56 patients (21). However, these results are conflicting since TMT measurement using brain CT was not related to functional outcomes in older patients with acute cerebral stroke in the analysis of Nozoe et al. (10). Of note, patients with dysphagia were excluded from this study, which could contribute to the negative finding since recent reports suggested that sarcopenia is one of the causes of post-stroke dysphagia (22, 23). Unfortunately, our analysis of cerebellar stroke patients did not include the analysis of dysphagia, which certainly should be addressed in future.

Regarding the role of TMT after cerebral ischemic stroke, we report for the first time that TMT, after acute cerebellar stroke, is a disease compromising only 1.5% of all ischemic strokes (5). Our study adds an important input of the rare cohort of cerebellar ischemic strokes, which is not studied in the abovementioned analysis. Low TMT was significantly associated with reduced outcomes at discharge and at 12 months.

### Limitations

Although our analysis shows the value of TMT in a sizable cohort of cerebellar stroke patients, our study faces some limitations. The validity of the cutoff values for identifying sarcopenia is still the subject of scientific discussion and could result in lower TMT values in comparison to our dichotomization. A further limitation is due to the fact that other factors not considered here could play a role in the clinical condition after 12 months. Close clinical monitoring with regular reevaluation should be considered in future prospective studies. The retrospective analysis of TMT prohibited the evaluation of anatomical–functional relationships. Furthermore, as this is a retrospective observational study, confounding, selection bias, and uncontrolled statistical error risk cannot be excluded. Hence, future validation of the association of TMT with prognosis in patients with cerebellar stroke is warranted.

## Conclusion

Since data on factors that influence recovery in patients with cerebellar ischemic stroke are rare, we propose the TMT assessment in cerebellar stroke as an objective parameter with prognostic value, novel for cerebellar stroke patients.

## Data availability statement

The original contributions presented in the study are included in the article/supplementary material, further inquiries can be directed to the corresponding author.

## Ethics statement

The studies involving humans were approved by Ethics Committee of the University Medicine Rostock, Germany (Identification number: A 2021-0112). The studies were conducted in accordance with the local legislation and institutional requirements. Written informed consent for participation was not required from the participants or the participants' legal guardians/next of kin in accordance with the national legislation and institutional requirements.

## Author contributions

DD and S-YW collected the data and wrote the first draft. FG supervised the manuscript. All authors supplied additional information, edited the manuscript, and contributed to the critical review and revision of the manuscript.
